# Role of gut microbiota and metabolomics in the lipid-lowering efficacy of statins among Chinese patients with coronary heart disease and hypercholesterolemia

**DOI:** 10.3389/fcimb.2024.1408581

**Published:** 2024-07-25

**Authors:** Lihua Hu, Boxian Hu, Long zhang, Yuhong Hu, Yali Zhang, Ruihang Zhang, Hongxi Yu, Dan Liu, Xiaolei Wang, Ouya Lin, Yanjun Gong, Yan Zhang, Cheng Li, Jianping Li

**Affiliations:** ^1^ Department of Cardiology, Peking University First Hospital, Beijing, China; ^2^ Institute of Cardiovascular Disease, Peking University First Hospital, Beijing, China; ^3^ School of Engineering Medicine of Beihang University and Key Laboratory of Big Data-Based Precision Medicine (Beihang University), Ministry of Industry and Information Technology of China, Beijing, China; ^4^ Department of Occupational and Environmental Health Sciences, School of Public Health, Peking University, Beijing, China; ^5^ State Key Laboratory of Vascular Homeostasis and Remodeling, Peking University, Beijing, China; ^6^ National Health Commission (NHC) Key Laboratory of Cardiovascular Molecular Biology and Regulatory Peptides, Beijing, China

**Keywords:** gut microbiota and hypercholesterolemia hypercholesteremia, statins, gut microbiota, metabolomics, biomarker

## Abstract

**Background:**

Statins, being the primary pharmacological intervention for hypercholesterolemia, exhibit a notable degree of interpatient variability in their effectiveness, which may be associated with gut microbiota. This study sought to identify the biomarkers for evaluating differences in statin efficacy.

**Methods:**

A quasi case-control study was conducted among participants with hypercholesterolemia and coronary heart disease taking rosuvastatin essential. According to the level of low density lipoprotein cholesterol (LDL-C), participants was divided into the “Up to standard” (US) group and the “Below standard” (BS) group. 16S rDNA sequencing and untargeted metabolomics were applied to detected the information of gut microbiota and related metabolites.

**Results:**

A total of 8 US and 8 BS group matched by age and sex were included in the final analysis. 16S rDNA sequencing results indicated that the characteristic strains of the US group were f-Eubacterium_coprostanoligenes and *g-Papillibacter*, while the characteristic flora of the BS group were o-C0119*, g-Pseudolabrys, s-Dyella-Marensis and* f-Xanthobacaceae. Metabolomic results suggested that the levels of chenodeoxycholic acid-3-β-D-glucuronide, 1-methylnicotinamide and acetoacetate in stool samples of the US group were significantly higher than those of the BS group. By identifying the differentially abundant bacterial taxa, the gut microbiota could modulate the efficacy of statins through producing enzymes involved in cholesterol metabolism.

**Conclusions:**

The findings suggest that the difference in statin efficacy may be related to gut microbiota strains that can produce short-chain fatty acids and secondary bile acids and affect the efficacy of statins by regulating the activities of cholesterol metabolite-related proteins. Metabolites related to short-chain fatty acids and secondary bile acids in the gut are expected to be biomarkers indicating the efficacy of statins.

## 1 Introduction

As we know, the prevalence of atherosclerotic cardiovascular disease (ASCVD) has persisted at an elevated and escalating rate, thus emerging as the foremost contributor to global mortality. An increasing researches have revealed that hypercholesterolemia, predominantly characterized by elevated levels of low-density lipoprotein cholesterol (LDL-C) in the bloodstream, constitutes a significant and modifiable risk factor for ASCVD (Sirtori, 2014; Raja et al., 2023). Presently, the incidence of hypercholesterolemia in China stands at 4.9%, with a persistent upward trend. Projections indicate that between 2010 and 2030, an estimated 9.2 million novel cardiovascular events attributable to hypercholesterolemia will manifest in China (Moran et al., 2010). Indeed, the accurate and efficient reduction of cholesterol levels holds immense significance in both the treatment and prevention of ASCVD.

Statins, being the foremost pharmacological agents employed in the management of hypercholesterolemia, hold significant prominence. Among these, rosuvastatin stands out due to its potent inhibitory effect on HMG-CoA reductase and pronounced hydrophilicity, thereby yielding favorable outcomes in LDL-C reduction (Lamb, 2020). However, it is crucial to acknowledge the considerable interindividual variations observed in the lipid-lowering efficacy of statins within the realm of clinical practice. Studies have demonstrated that patients administered identical statin types and dosages can exhibit a wide range of reductions in blood LDL-C levels, spanning from a modest 5% to a remarkable 70% (Mangravite et al., 2006; Barber et al., 2010; Postmus et al., 2014), which may be related to genes that affect statin metabolism. However, despite actively controlling for these known influencing factors, there are still significant differences in statin efficacy. We speculate that it may be related to the gut microbiota.

The gut is home to a vast array of microorganisms that contribute to various metabolic processes. The gut microbiota plays a crucial role in pharmacokinetics, as the use of drugs can disrupt the composition of the gut microbiota. Research by Moore et al. has shown that the gut microbiota is a key determinant of individual differences in drug reactions, as it influences the absorption, distribution, metabolism, and elimination of drugs (Sousa et al., 2008). Furthermore, the efficacy of drugs can be influenced by the gut microbiota. Studies have demonstrated that antibiotics can interfere with the metabolism of lovastatin in the human body, thereby affecting its pharmacokinetics and effectiveness (Yoo et al., 2014). The concentration of simvastatin in the bloodstream is strongly correlated with the levels of secondary bile acids, which are produced through microbial 7-α-dehydroxylation in the human gut (Kaddurah-Daouk et al., 2011). Patients who respond well to atorvastatin treatment have been found to have higher gut microbial diversity. Similarly, a group of individuals who experience a positive treatment effect (LDL-C < 3.64 mmol/L after 4 weeks of treatment) have a higher proportion of firmicutes in their gut microbiota compared to those with a less favorable treatment outcome (LDL-C > 3.64 mmol/L after 8 weeks of treatment) (Liu et al., 2018).

Statins are a class of drugs that are absorbed through the gastrointestinal tract and enter the systemic circulation by crossing the small intestinal wall. They exert their pharmacological effects by reducing cholesterol levels in the body (Hirota et al., 2020). Interestingly, statins can also have an impact on the composition of the gut microbiota in patients, and conversely, the gut microbiota can interact with statins (Vourakis et al., 2021). The relationship between statins and the gut microbiota is an area of growing interest, as it has been found to be closely associated with the lipid-lowering effects of statins. By studying the gut microbiota of patients, it may be possible to achieve personalized and precise regulation of blood lipids. However, it is important to note that there is currently limited research in this field. Therefore, this study aims to fill this gap by conducting a quasi case-control study analysis to explore the relationship between the lipid-lowering efficacy of statins and the gut microbiota. This research will contribute to our understanding of how the gut microbiota influences the effectiveness of statin therapy and may provide valuable insights for personalized lipid-lowering strategies.

## 2 Methods

### 2.1 Study design and population

All patients enrolled in this study were hospitalized in the Department of Cardiology, Peking University First Hospital in Beijing, China. Inclusion criteria are as follows: (1) Age ≥ 18 years old; (2) Patients with hypercholesterolemia diagnosed according to the Guidelines for the Prevention and Treatment of Adult Dyslipidemia in China (2016); (3) Patients were diagnosed with coronary heart disease by coronary angiography; (4) No health products or drugs affecting the gut microbiota was used for nearly one month; (5) Currently taking 10mg rosuvastatin (codeine, AstraZeneca Pharmaceutical Co., Ltd.) once a day for more than 3 months and not taking other lipid-lowering drugs. Exclusion criteria are as follows: (1) Use of antibiotics and dairy products in recent one month; (2) Long-term use of steroid hormones, thyroid hormones, contraceptives; (3) Chronic gastrointestinal diseases; (4) Adrenal cortex function decreases; (5) Hypothyroidism; (6) Current or past drug abuse. Subjects who met all the inclusion criteria and did not meet any of the exclusion criteria were included in the study. According to 2019 ESC/EAS Guidelines for the management of dyslipidemias, 2021 ESC Guidelines on cardiovascular disease prevention in clinical practice and 2023 Chinese Guidelines for the management of blood Lipid, patients with established CHD who are at very-high risk, an LDL-C goal of <1.8 mmol/L (<70 mg/dL) are recommended. Therefore < patients were divided into two groups: “Up to Standard” group (US group) (LDL-C <1.8 mmol/L) and “Below to Standard” group (BS group) (LDL-C ≥1.8 mmol/L). According to the same gender and age (± 3 years), the qualified group and the non-qualified group were matched. This study matched US group with an equal number of BS group (patients without stroke) for age ± 3 years and sex. Referring to previously published papers on small sample studies of gut microbiota, a total of 16 patients were included (8 participants in US group; 8 participants in BS group). The study was approved by the Ethics Committee of the Institute of Biomedicine, Peking University First Hospital, China. All participants signed an approved written consent after it was explained to them.

### 2.2 Stool samples and DNA extraction

Stoll sampling procedures followed the requirements of the German Sarstedt fecal collection system. After feces samples were obtained from patients, feces samples were mixed evenly with a sterile spatula, loaded 1 g into 12 ml sterile refrigerated tubules, labeled and numbered, and stored in a -20°C refrigerator. Samples were shipped to the laboratory within 24 h for storage at -80°C. Using CTAB/SDS (cetyltrimethylammonium bromide/sodium lauryl sulfate) to extract total genomic DNA from samples. The total DNA from samples was extracted using CTAB/SDS method. DNA concentration and purity was monitored on 1% agarose gels. According to the concentration, DNA was diluted to 1 ng/µL using sterile water.

### 2.3 16S rDNA sequencing

The 16S rDNA gene in V3-V4 region was amplified by primer and DNA sample with the concentration of 1 ng/µL. The primer sequences used are as follows (5’-3’ from left to right): 341F: CCTAYGGGRBGCASCAG; 806R: GGACTACNNGGGTATCTAAT. A PCR system was prepared with various reagents and materials according to the following proportions: 15 µL of Phusion High-Fidelity PCR Master Mix (New England and Biolabs, UK), 0.2 µL of primers DNA 10 ng of target DNA. The PCR cycle conditions are set as follows: denaturation at 98-°C for 1 min, then cycle at 98-°C (10 s), 50-°C(30 s) and 72-°C (30 s) for 30 times, and finally extend at 72-°C for 5 minutes.

The amplified product was mixed with equal volume of 1 × loading buffer (containing SYB green) and detected by 2% agarose gel electrophoresis. Electrophoresis parameters are: agarose gel concentration: 2%; Voltage: 80 v; Electrophoresis time: 40 min. After the detection, PCR products from different samples were mixed in equal amounts and purified using Qiagen Gel Extraction Kit (Qiagen, Germany). Sequencing library was generated by Illumina Truseq DNA PCR-free library preparation kit (Illumina, USA), quantified by Qubit2.0fluorometer (Thermo Science, USA) and Agilent Bioanalyzer 2100 system (Agilent, China). Finally, 250 base-pair end readings were sequenced on the Illumina NovaSeq platform (Illumina, the US). Sequencing and data pretreatment were completed in Beijing Nuohe Zhiyuan Company.

### 2.4 Metabolites extraction

The stool samples (100 mg) were individually grounded with liquid nitrogen and the homogenate was resuspended with prechilled 80% methanol by well vortex. The samples were incubated on ice for 5 min and then were centrifuged at 15000 g, 4°C for 20 min. 400 μL supernatant was diluted to final concentration containing 53% methanol by LC-MS grade water. The samples were subsequently transferred to a fresh Eppendorf tube and then were centrifuged at 15000 g, 4°C for 20 min. Finally, the supernatant was injected into the LC-MS/MS system analysis (Want et al., 2013).

### 2.5 Non-targeted metabolomics detection

UHPLC-MS/MS analyses were performed using a Vanquish UHPLC system (ThermoFisher, Germany) coupled with an Orbitrap Q Exactive TM HF mass spectrometer or Orbitrap Q Exactive

TMHF-X mass spectrometer (Thermo Fisher, Germany) in Novogene Co., Ltd. (Beijing, China). Samples were injected onto a HypesilGoldcolumn (C18) using a 12-min linear gradient at a flow rate of 0.2 mL/min . The eluents for the positive and negative polarity modes were eluent A (0.1% FA in Water) and eluent B (Methanol). The solvent gradient was set as follows: 2% B, 1.5 min; 2-85% B, 3 min; 85-100% B, 10 min;100-2% B, 10.1 min;2% B, 12 min. Q Exactive TM HF mass spectrometer was operated in positive/negative polarity mode with spray voltage of 3.5 kV, capillary temperature of 320°C, sheath gas flow rate of 35 psi and aux gas flow rate of 10 L/min, S-lens RF level of 60, Aux gas heater temperature of 350°C. min.

### 2.6 Covariates

Covariates included sex, age (years), body mass index (BMI, kg/m^2^), current smoking, current drinking, history of hypertension, history of diabetes, antihypertensive drugs, glucose-lowering drugs, white blood cell (WBC), high sensitivity c reactive protein (hsCRP, mg/L), total cholesterol (TC, mmol/L), triglycerides (TG, mmol/L), high density lipoprotein cholesterol (HDL-C, mmol/L), uric acid (UA, μmol/L) and estimated glomerular filtration rate (eGFR, mL·min^−1^.1.73 m^−2^).

### 2.7 Statistical analyses

Data are presented as median (*P*_25_-*P*_75_) for continuous variables and as frequency (%) for categorical variables. Differences in baseline characteristics between two groups were compared using generalized Mann-whitney U tests for continuous variables and chi square tests for categorical variables.

Based on the returned operational taxonomic unit (OTU) table and species annotation results, follow-up data analysis was carried out. One sorting operation unit represented one strain. Draw venn plot and petal plot in RStudio to visually display OTU annotation. Took the top 10 microorganisms with the highest relative abundance at phylum, family and genus level as cumulative bar graph in SPSSAU, and analyze the difference of the relative abundance of the top 10 relative abundances of bacteria at different classification levels.

RStudio was used to calculate the α diversity index of the two groups of samples, and the difference of α diversity between the two groups was judged by Mann-Whitney test in SPSSAU (https://spssau.com). The α diversity used in this study include Goods-coverage, Chao1, Ace, Shannon and Simpson indices. The differences of microbial composition between the two groups were analyzed from three aspects: sequencing depth, microbial abundance and microbial diversity. Using RStudio to analyze β diversity based on Bray-curtis distance matrix. Calculated the β diversity of the two groups of samples, and analyzed whether the difference is significant by Mann-Whitney test in SPSSAU. Completed principal co-ordinates analysis (PCoA), principal component analysis (PCA) and non-metric multidimensional scaling (NMDS) to reflect the difference between the samples within and between groups. Drawn UGPMA cluster plot to show the results of microbial β diversity analysis among groups visually. Using LefSe online analysis website (http://huttenhower.sph.harvard.edu/galaxy) to select representative species with differences between groups.

Using KEGG database (https://www.genome.jp/kegg/pathway.html), HMDB database (https://hmdb.ca/metabolites) and LIPIDMaps database (http://www.lipidmaps.org) to annotate the identified metabolites. Metabolomics data processing software metaX was used to preprocess the original data. The data preprocessing was completed by Beijing Nuohe Zhiyuan Company. The results obtained in positive and negative ion modes were combined. RStudio and SPSSAU were used for data analysis and drawing. PCA analysis and partial least squares discriminant analysis (PLS-DA) were used to reflect the differences of metabolites within and between groups. The variable influence on projection (VIP) value of each metabolite was obtained from the data obtained by PLS-DA method. The fold change (FC) of each metabolite between the two groups was calculated according to the measured content of each metabolite in each sample. The statistical significance difference of each metabolite between the two groups was calculated based on T-test. The metabolites with VIP > 1.4 and *P*-value < 0.05 and FC > 2 or FC < 0.5 were considered to be differential metabolites. Volcano plots were used to filter metabolites of interest which based on log2(FC) and -log10(p-value) of metabolites by ggplot2 in R language. The standard score (Z-score) of the differential metabolites ranked as Top30 according to *P*. The area under the receiver operating characteristic curve (ROC) of different metabolites was calculated in SPSSAU, which was used as a reference standard to determine biomarkers. According to the specific biological significance of different metabolites, combined with AUC value, biomarkers were determined. Metabolic pathway enrichment analysis of differential metabolites was performed based on the KEGG database. Metabolic pathways with *P* < 0.05 were significantly enriched by differential metabolites.

All the analyses were performed using the statistical package R (http://www.R-project.org, Te R Foundation. A 2-tailed *P*<0.05 was considered to be statistically significant.

## 3 Results

### 3.1 Baseline characteristics of study participants

A total of 16 patients were included (8 participants in US group; 8 participants in BS group). As shown in **Table 1**, compared with the US group, participants in BS group had higher TC and LDL-C levels. No significant differences were found between the two groups in terms of sex, gender, smoking, drinking, WBC, hsCRP, TG, HDL-C, FBG and eGFR.

**Table 1:** General clinical comparison of US group and BS group.

Variables^*^	US (n=8)	BS (n=8)	*P* value
Male, n(%)	6 (75.00)	6 (75.00)	1.000
Age	63.50 (60.50, 70.20)	64.50 (53.20, 71.00)	0.958
BMI	24.62 (22.89, 25.41)	25.15 (22.44, 26.49)	0.529
Smoking, n (%)	4 (50.00)	4 (50.00)	1.000
Drinking, n (%)	3 (37.50)	3 (37.50)	1.000
History of hypertension, n (%)	6 (75.00)	4 (50.00)	0.170
History of diabetes, n (%)	2 (25.00)	5 (62.50)	0.315
Antihypertensive drugs, n (%)	6 (75.00)	4 (50.00)	0.170
Glucose-lowering drugs, n (%)	1 (12.50)	3 (37.50)	0.569
WBC (×10^9^/L)	5.10 (4.62, 6.15)	5.85 (4.82, 6.92)	0.247
hsCRP (mg/L)	0.91 (0.39, 1.94)	1.28 (0.76, 1.84)	0.372
TC (mmol/L)	3.26 (3.02, 3.61)	4.56 (4.07, 5.21)	0.002
TG (mmol/L)	1.02 (0.67, 2.06)	1.34 (0.88, 1.75)	0.674
HDL-C (mmol/L)	1.04 (0.95, 1.33)	1.18 (0.99, 1.48)	0.343
LDL-C (mmol/L)	1.64 (1.37, 1.74)	2.52 (2.22, 2.98)	0.001
FBG (mmol/L)	7.08 (5.77, 9.36)	7.17 (4.94, 9.75)	0.753
UA (μmol/L)	360.00 (328.25, 399.00)	390.50 (331.50, 417.75)	0.495
eGFR (ml/min/1.73m^3^)	71.04 (64.36, 93.29)	85.52 (71.01, 97.83)	0.345

BMI, body mass index; WBC, white blood cell; hsCRP = TC, total cholesterol; TG, triglycerides; HDL-C, high density lipoprotein cholesterol; FBG, fasting blood glucose; UA, uric acid; eGFR, estimated glomerular filtration rate.

### 3.2 Sequencing quality control

**Figure 1:**
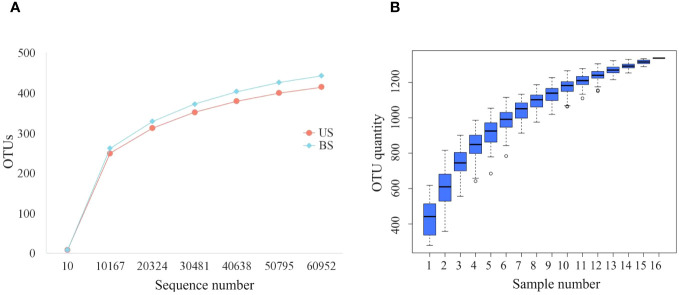
Sequencing quality control up to standard. **(A)** Dilution curves of US group and BS group; **(B)** Species accumulation box.

After processing the data, we get 77402-95293 initial sequence. It could be seen from the dilution curve (**Figure 1A**) that with the increase in the number of sequences, the increase rate gradually slowed down. When the number of sequences increased from 50815 to 60976, the curve was nearly flat and entered a plateau period, indicating that the sequencing depth was appropriate. As shown in the cumulative box plot of species (**Figure 1B**), when the number of samples was 15 or 16, the number of OTUs had hardly increased, and the curve became smooth, indicating that the number of 16 samples was sufficient, which could reflect the gut microbiota of patients in the group more comprehensively and could be used for subsequent analysis.

**Figure 2:**
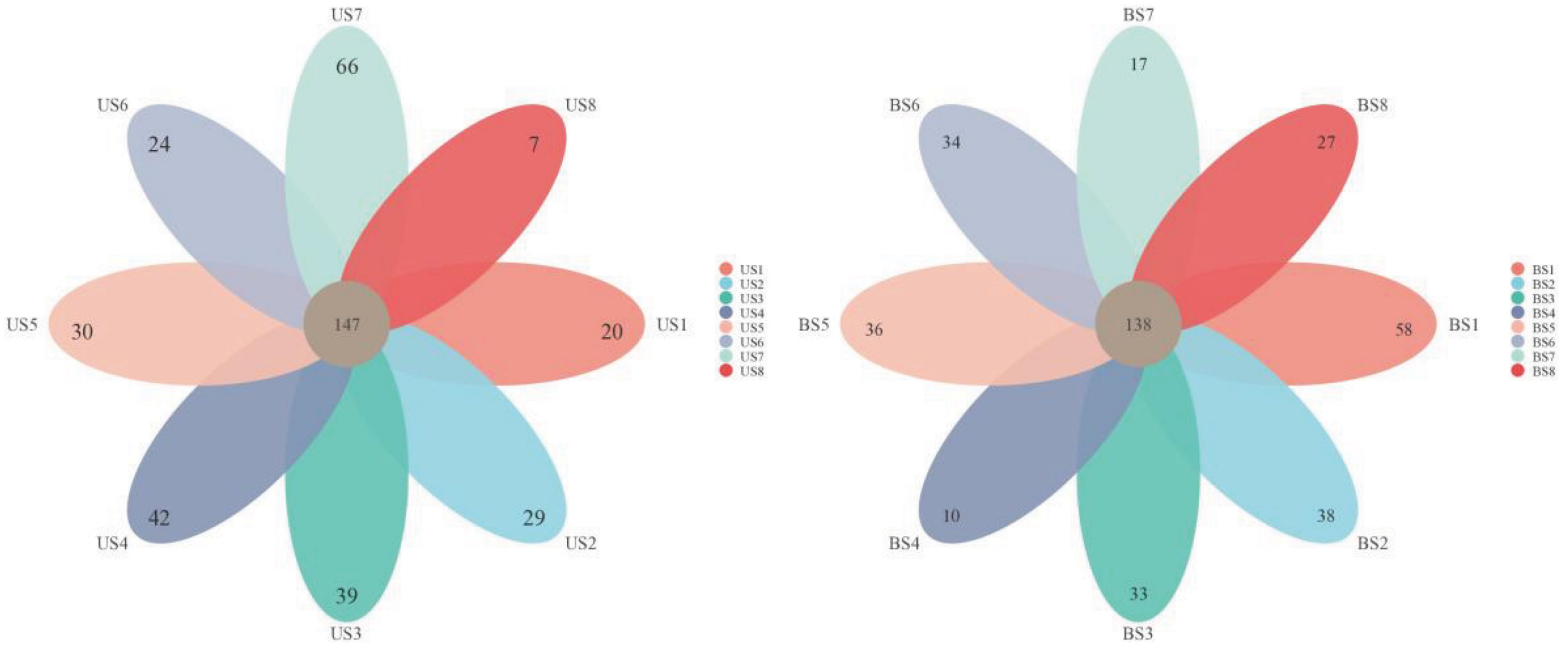
Venn plot and petal plots for OTUs between the US and BS groups.

After the sequence was annotated, the OTU clustering map was obtained. A total of 1,338 OTUs were detected, of which 829 were detected in both US and BS groups. There were 216 OTUs and 293 OTUs specific to the US group and the BS group, respectively. The OTUs annotation within the US and BS groups was shown in the petal plot in **Figure 2**. In the US group, 148 OTUs could be detected in all samples. Although sample US7 contained more non-common OTUs, the overall trend showed that most OTUs were common to all samples. In the BS group, 136 OTUs were detectable in all samples, and the overall trend remains that most OTUs were common within the group.

### 3.3 Types of gut microbiota

**Figure 3:**
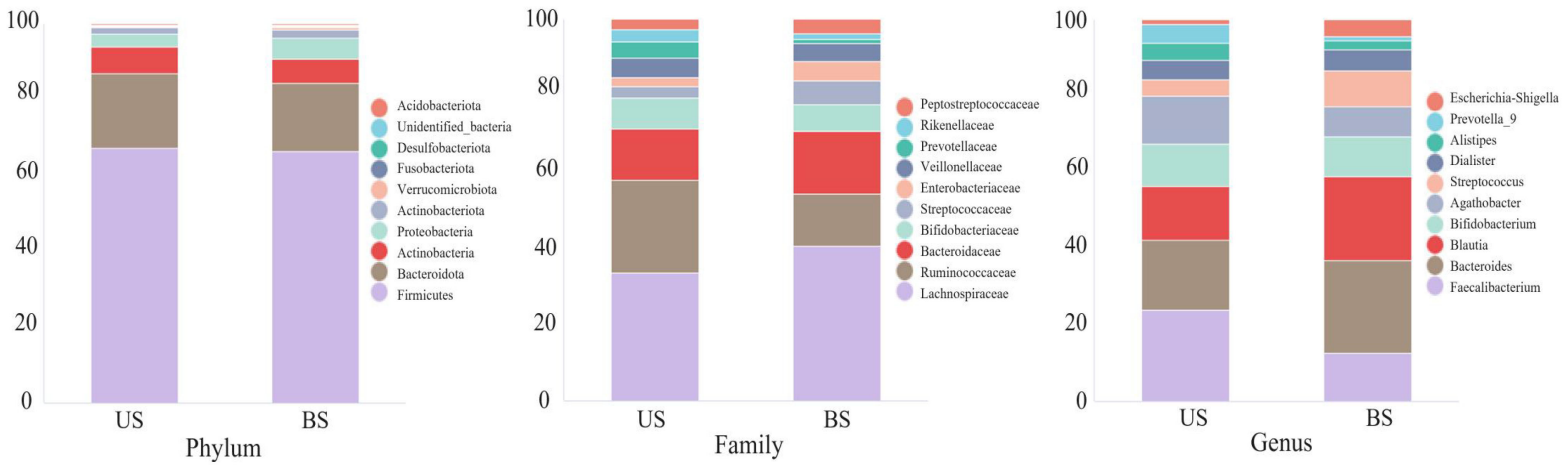
Top ten relative abundances of bacteria at the phylum, family and genus levels.

As shown in **Figure 3**, the distribution differences among the 10 OTUs with the highest relative abundance at the phylum, family and genus levels were displayed. At the phylum level, there was no significant difference in the relative abundance of the microflora between the two groups. At the family level, the *Rumen Bacteroides* and *Bifidobacterium* families accounted for more proportion in the US group than in the BS group, while the relative abundance of *Chaetomium* and *Bacteroides* was lower than that in the BS group. At the genus level, the levels of *Faecalibacterium* and *Bifidobacterium* in the US group were higher than those in the BS group, and the levels of *Bacteroides* and *Blautia* were lower than those in the BS group.

### 3.4 Gut microbiota composition comparison

**Figure 4:**
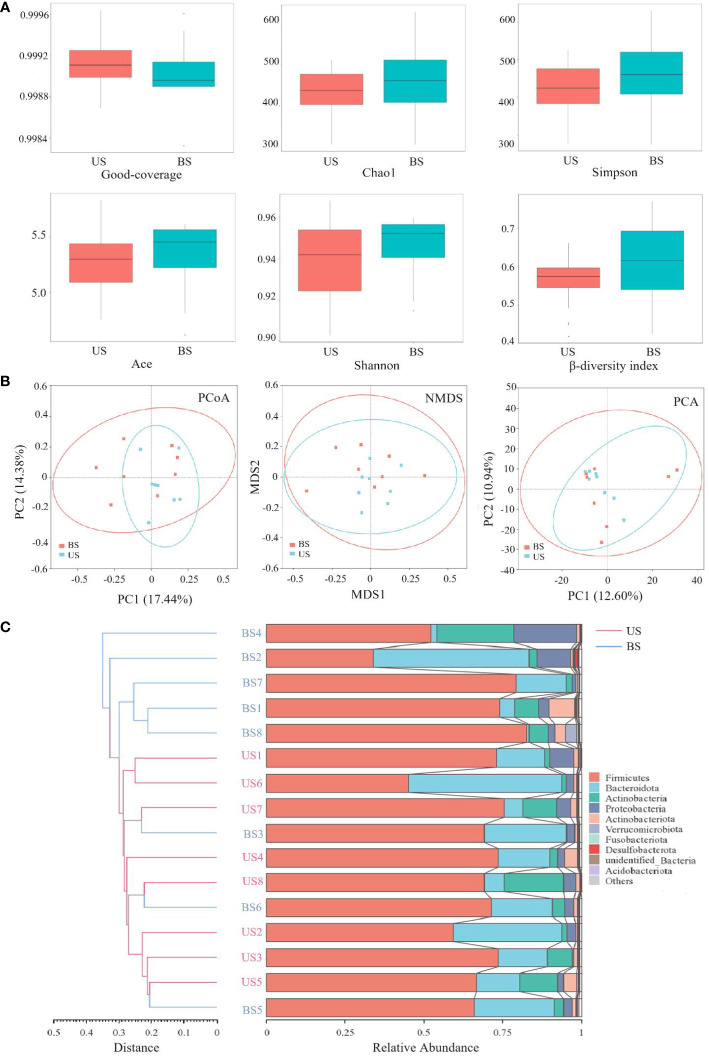
Comparison of flora diversity and abundance between the US and BS groups. **(A)** α and β diversity between groups. **(B)** Two-dimensional diagram of group difference analysis. **(C)** UPGMA cluster plot obtained by Bray-curtis algorithm.

The comparison of α-diversity between two groups was shown in **Figure 4A**. There was no significant difference in the Good-coverage index between the US group and the BS group (*P* = 0.507), indicating that the results of the tests in each group reflected the actual situation of samples approximately. There was no significant difference in Chao1 index (*P* = 0.561) and Ace index (*P* = 0.508), indicating that there was no significant difference in the abundance of gut microbiota between the two groups. There was no significant difference between the Shannon index (*P* = 0.816) and Simpson index (*P* = 0.582), indicating that there was no significant difference in microbial community diversity between the two groups. Based on the Bray-curtis distance matrix, Mann-Whitney test, PCoA analysis and NMDS analysis were carried out on the samples of the US group and the BS group. Mann-Whitney test was used to test the β-diversity index between the US group and the BS group (**Figure 4A**), and the result was significant (*P*=0.036). PCoA and NMDS results were not significantly differentiated between the two groups of samples (**Figure 4B**). PCA results showed that there was significant aggregation of the US group (**Figure 4B**), and only individual samples overlapped with those of the BS group. On the overall level, there was a significant separation trend between the two groups. The UPGMA results (**Figure 4C**) showed that the samples of the US group were basically at the bottom of the clustering plot, while the samples of the BS group were basically at the upper of the clustering plot, and some samples of the BS group were interspersed among the samples of the US group. However, the overall results showed that the samples within the group are similar, and the groups could be generally distinguished. This phenomenon indicated that there were differences in the gut microbiota diversity and microbiota abundance between the US group and the BS group to a certain extent.

**Figure 5:**
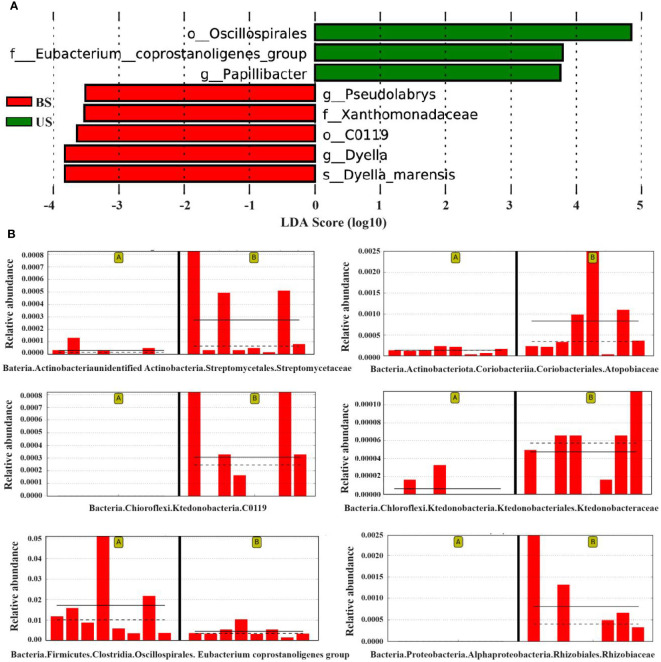
LefSe analysis found the difference between groups. **(A)** LDA score map of differential species. **(B)** Relative abundance map of different strains. On the left of each relative abundance map are the samples from the substandard group and on the right are the substandard group. The bar chart shows the relative abundance of the differential species in each sample linear measure. The solid lines represent the average relative abundance of differential species in this group, and the dashed line represents the median relative abundance of differential species in this group.

The LefSe (Linear Discriminant Analysis Effect Size) analysis was conducted to select the differential strains with distinguishing effect between the two groups. The LDA Score >3.5 was set as the threshold, and 8 differential strains at different classification levels were screened (**Figure 5A**). The results of the lowest classification level were reserved for the differential strains, and 6 different differential strains were obtained in total, the dominant strains in US were f-Eubacterium_coprostanoligenes_group, and *g-Papillibacter*. The dominant strains in the BS group were o-C0119, *g-Pseudolabrys*, *s-Dyella-marensis*, and f-Xanthomonadaceae. The species that showed significant differences between the two groups were o-C0119 (*P*=0.011), *f-Eubacterium_coprostanoligenes_group* (P = 0.016), and *g-Pseudolabrys* (*P* = 0.027) (**Figure 5B**).

### 3.5 Results of untargeted metabolomics

**Figure 6:**
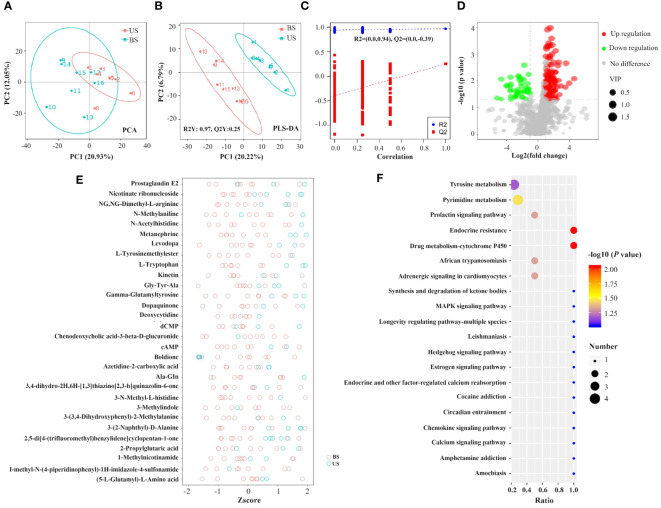
Screening of differential metabolites and analysis of metabolic pathways. **(A)** PCA plot. **(B, C)** PLS-DA plot. In the left scatter plot, R2Y represents the interpretation rate of the model, Q2Y is used to evaluate the predictive power of the PLS-DA model, and Q2Y is well established when R2Y is larger than Q2Y. In the right rank test plot, the abscissa represents the Y of the randomized group and the original group Y correlation, the ordinate represents the scores in R2 and Q2. **(D)** Volcanic map of differential metabolites. Green circles represent differential metabolites significantly downregulated in the standard group compared to the substandard group, while red circles are significantly up-regulated in the standard group compared to the substandard group. The size of the circle is positively correlated with its represented metabolite VIP values. The horizontal axis represents the fold difference of metabolites between the two groups, and the vertical axis represents the significant degree of the difference. **(E)** Differential metabolite Zscore score plot. **(F)** KEGG pathway function annotation bar graph of positive ion compounds: the X-axis represents the number of metabolite annotations, and the Y-axis represents the annotated KEGG pathway.

As shown in the PCA plot (**Figure 6A**), the US group and the BS group samples were significantly separated, and there was significant difference between the two groups of metabolites. PLS-DA plot (**Figures 6B, C**) showed that the US group and the BS group were significant distinguished, and the model could effectively distinguish the two groups of samples. Screening based on the conditions of VIP > 1.4; FC > 2 or FC < 0.5 identified 104 differential metabolites, of which 80 metabolites were significantly up-regulated and 24 metabolites were significantly down-regulated in the US group compared to the BS group. The differential metabolite information was plotted as a volcano diagram (**Figure 6D**), which can visualize the overall distribution of differential metabolites in the two groups. Zscore was calculated according to the formula Z = (x-μ)/σ. x represents the specific content of a metabolite, μ represents the mean value of the metabolite, and σ refers to the standard deviation. The relative concentrations of the top 30 differential metabolites were plotted as Z-score (**Figure 6E**) in descending order of *P*-value. Among the 104 differential metabolites, 35 with an AUC value was greater than 0.9, and 66 with an AUC value was within the range of 0.7-0.9.

A total of 57 KEGG enrichment pathways were identified (**Figure 6F**), including 6 significant pathways as follows: Endocrine resistance (*P*=0.009), drug metabolism -cytochrome P450 (*P*=0.009), pyrimidine metabolism (*P*=0.035), adrenergic signaling in cardiomyocytes (*P*=0.048), prolactin signaling pathway (*P*=0.048), and African trypanosomiasis (*P*=0.048).

## 4 Discussion

Screening for differential flora holds significant potential in guiding healthcare professionals towards more rational drug usage, ultimately leading to precise control of blood lipid levels. However, it is important to acknowledge that the current understanding of screening for differential flora is still incomplete and imperfect. In our study, we identified six distinct strains that exhibited differential abundance between the two groups. The representative strains in the US group were *f-Eubacterium-coprostanoligenes* and *g-Papillibacter*, *while o-C0119*, *g-Pseudolabrys*, *s-Dyella-Marensis*, and *f-Xanthobacaceae* were representative strains in the BS group. The representative strains in the US group were found to assist in the lipid-lowering effects of rosuvastatin through various mechanisms. They inhibited cholesterol synthesis and facilitated its elimination. For instance, *f-Eubacterium-coprostanoligenes* secretes cholesterol dehydrogenase ECOP170, which aids in the elimination of cholesterol through feces (Koppel et al., 2017; Kenny et al., 2020). On the other hand, g-Papillibacter produces butyric acid (Konikoff and Gophna, 2016), which acts as a short-chain fatty acid (SCFA) that inhibits hepatic cholesterol synthesis and promotes the redistribution of cholesterol from the blood to the liver (Taylor and Williams, 1998). These mechanisms contribute to the overall lipid-lowering effects observed in the US group.

The representative strains of the BS group were all Gram-negative. Gram-negative bacteria have an outer layer of lipopolysaccharide components called endotoxin, which can cause endotoxemia, resulting in local inflammation and metabolic disorders that increase adipose tissue production (Pussinen et al., 2022). It was deduced that endotoxin had a negative effect on the lipid metabolism in patients of this group, and at the same time, weakened the efficacy of rosuvastatin. By employing KEGG pathway enrichment analysis, we uncovered a noteworthy enrichment of differential metabolites within the endocrine resistance pathways and drug metabolism-cytochrome P450 (CYP450) pathways, highlighting distinct metabolic profiles between the two investigated groups. Notably, the metabolism-CYP450 pathway exhibited a significant enrichment of differential metabolites, including Valproic acid (VPA) and 2-PGA, which were found to be present in substantially higher quantities in fecal samples from the US group compared to those from the BS group. Intriguingly, the KEGG pathway plot illustrates the human metabolism of valproic acid, wherein it undergoes biotransformation by CYP2C9 enzymes belonging to the cytochrome P450 family, ultimately leading to the formation of 2-PGA, the final metabolite in this pathway.

Notably, statins, irbesartan, and loratadine are among the pharmaceuticals that undergo metabolism mediated by the CYP450 family. Shah et al., has shed light on the influence of patients’ drug metabolism capacity on their responses to medications. Genetic variations in the expression and activity of drug metabolism enzymes can significantly alter the pharmacological properties of drugs, ultimately impacting their efficacy and potential toxicity (Shah and Smith, 2015). In the context of metabolism, CYP2C9 enzymes, which are part of the CYP450 family, play a crucial role in the metabolism of rosuvastatin. Lin et al., revealed that patients with a mutant CYP2C9 exhibited a notably superior LDL-C lowering effect after rosuvastatin administration compared to individuals with the wild-type CYP2C9. This suggests that CYP2C9 gene polymorphisms contribute to distinct CYP2C9 enzyme activities and metabolic capabilities towards rosuvastatin, consequently influencing the lipid-lowering effect of rosuvastatin in patients with hypercholesterolemia (Lin et al., 2015), which serves as a reminder that the activities of drug-metabolizing enzymes in patients themselves exert a certain influence on the lipid-lowering efficacy of statins. Considering the higher detection of the VPA final metabolite, 2-PGA, in fecal samples from patients in the US group, it can be speculated that patients in this group may have absorbed less VPA. Our observation suggests that the CYP2C9 enzyme activity primarily responsible for VPA metabolism in the liver of patients in the US group might be higher compared to that in the BS group. Consequently, it is reasonable to estimate that the diminished efficacy of rosuvastatin in patients within the substandard group may be attributed to their lower CYP2C9 enzyme activity and reduced metabolic capacity (Lin et al., 2015).

Moreover, Differential metabolites also enriched in the endocrine resistance pathway included estradiol and cAMP. The gut microbiota plays an important role in the regulation of estrogen in humans, and Plottel et al., showed that some intestinal microorganisms are able to secrete the enzyme β-glucuronidase, which helps to convert estrogen from the bound form to the unbound form, thus allowing the unbound form of estrogen to re-enter the circulation, and decreasing the activity of the enzyme leads to an increase in the fecal excretion of estrogen (Cullin et al., 2021; Qi et al., 2021). Therefore, the level of estradiol in fecal samples was lower in the US group than in the BS group, and the level of estrogen in circulating blood was higher than in the BS group. Higher circulating blood estrogen concentrations may prevent obesity, metabolic syndrome, and cardiovascular disease (Franasiak and Scott, 2015).

Estradiol has the ability to activate adenylate cyclase (AC) through the G protein-coupled estrogen receptor 1 (GPER1) (Yasar et al., 2017). This activation leads to an increase in intracellular cAMP levels through the phosphatase catalysis of AC. Subsequently, the cAMP-PKA pathway is activated, which in turn promotes cAMP-mediated transcriptional activity. This signaling pathway has been implicated in various cellular processes. Cannon et al. demonstrated that the efficacy of statins is limited by a compensatory increase in 3-HYDROXY-3-methylglutamyl-CoA Reductase (HMGCR), the enzyme targeted by statins. However, if the compensatory increase in HMGCR can be inhibited, the efficacy of statins can be further improved (Cannon et al., 2015). The promoter region of the HMGCR gene contains a binding site for the cAMP response element (CRE), which can interact with the cAMP response element-binding protein (CREB) to regulate the transcription of the HMGCR gene (Paul et al., 2022). Furthermore, cAMP can activate protein kinase A (PKA), which phosphorylates the regulatory subunit of protein phosphatase 2A (PP2A) (Leslie and Nairn, 2019). This phosphorylation event leads to the release of PP2A, preventing the dephosphorylation and reactivation of HMGCR. Therefore, higher levels of cAMP can enhance the activity of the PKA pathway, inhibit the activation of HMGCR, and reduce the impact of the compensatory increase in HMGCR on the lipid-lowering efficacy of statins. Based on these findings, the lipid-lowering efficacy of rosuvastatin was found to be superior in the compliance group compared to the non-compliance group. This may be attributed to the higher level of cAMP in the compliance group, which improves the activity of the PKA pathway, inhibits HMGCR activation, and mitigates the impact of the compensatory increase in HMGCR on the effectiveness of rosuvastatin. Valproic acid is a short-chain fatty acid. Since the human body cannot synthesize VPA on its own and the enrolled patients did not have a history of medication use, we hypothesized that VPA is synthesized by intestinal microorganisms. However, unlike short-chain fatty acids such as butyric acid, VPA can affect lipid homeostasis by inhibiting the activation of AMP-activated protein kinase (AMPK), which promotes insulin resistance and oxidative stress (Duarte et al., 2021).

Based on the biological significance and area under the curve (AUC) values of the differential metabolites, three potential predictive biomarkers were identified: chenodeoxycholic acid-3-β-D-glucuronide, 1-Methylnicotinamide, and Acetoacetate. The levels of chenodeoxycholic acid-3-β-D-glucuronide detected in stool samples from patients in the US group were significantly higher than those in the BS group. Chenodeoxycholic acid is metabolically regulated in the liver and acts as a receptor for bile acids. Previous studies have demonstrated that the farnesoid X receptor (FXR) can activate the UGT2B4 gene, which is involved in the glucuronidation of bile acids (Bock, 2012). This process ultimately leads to a decrease in cholesterol levels in the body (Afonso et al., 2018). Therefore, the higher levels of chenodeoxycholic acid-3-β-D-glucuronide in the US group may indicate enhanced bile acid metabolism and excretion, resulting in a better lipid-lowering effect compared to the BS group after statin treatment. Levels of 1-methylnicotinamide detected in feces samples from patients in the US group were significantly higher than those in the BS group. Studies have shown that NNMT plays a role in reducing lipid levels in the body by modulating the expression of peroxisome proliferator-activated receptor (PPARα) (Sharma et al., 2015). Mice with a knockout of the NNMT gene have been found to have significantly higher serum cholesterol levels and lower hepatic PPARα expression. The higher level of 1-methylnicotinamide detected in the feces samples of the US group suggests that the expression level of NNMT in the liver of patients in this group is higher compared to the BS group. This higher expression level of NNMT may be more conducive to cholesterol metabolism, thereby enabling rosuvastatin to exert better lipid-lowering efficacy. Similarly, levels of acetoacetate detected in fecal samples from patients in the US group were significantly higher than those in the BS group. Acetoacetate is involved in three KEGG enrichment pathways: propionate metabolism, butyrate metabolism, and ketone body synthesis and degradation. Studies have shown that acetoacetate can down-regulate the gene transcription of ApoB100, ApoE, and LDL-R, which are related to LDL-C assembly (Hwang et al., 2022). This down-regulation inhibits the assembly of VLDL in bovine hepatocytes, contributing to the reduction of blood LDL-C levels. Additionally, it has been mentioned previously that intestinal microorganisms can regulate blood cholesterol levels by secreting short-chain fatty acids such as propionic acid and butyric acid. The higher levels of acetoacetate in the US group may indicate a stronger function of intestinal microorganisms in producing propionic acid and butyric acid, which is more conducive to reducing blood LDL-C levels. This, in turn, may help improve the lipid-lowering efficacy of rosuvastatin.

According to the analysis of gut microbiota, there was a difference in the microbiota capable of secreting short chain fatty acids between the two groups. For example, a higher proportion of *Bifidobacteriaceae* can be observed in the treatment group that meets the efficacy standards. *Bifidobacteriaceae* can produce acetic acid and propionic acid, while *rumen microbiota* can produce butyric acid; moreover, the representative bacterial species selected by the LefSE algorithm for the efficacy standard group, *Papillobacter genus*, is also a butyrate producing bacterium. These short chain fatty acids can inhibit the activity of HMGR, promote upregulation of LDL-R expression (Pushpass et al., 2021), and have a synergistic effect with statins, helping to reduce patient blood LDL-c levels. The metabolomics also showed an increase in acetyl acetate, a metabolite of the above short chain fatty acids. The findings suggest that more of the above short chain fatty acids maybe involved in the BS group. In addition to short chain fatty acids, the gut microbiota can also regulate human metabolism by producing secondary bile acids. Gut microbiota can ultimately affect cholesterol metabolism by affecting the activity of FXR protein. Previous study have confirmed that some secondary bile acids produced by gut microbiota could act as inhibitors of FXR proteins, and there were also secondary bile acids that could act as FXR protein stimulants (Wahlström et al., 2016). Lithocholic acid is a stimulant of FXR protein, while taurine β - rhamnocholic acid could inhibit the activity of FXR protein (Pushpass et al., 2021). The differences in the composition of gut microbiota between the two groups resulted in the differences in the composition of secondary bile acids produced by the microbiota, ultimately affecting the efficacy of statin drugs.

This study provides a preliminary understanding of the biological mechanism by which gut microbiota influences the lipid-lowering effect of rosuvastatin. It lays the foundation for further individualized treatment of hypercholesterolemia. However, the study has several key limitations. First, it was a cross-sectional study, the LDL-C levels and fecal samples before taking statins were not collected. Second, the sample size was small. Third, Additionally, this study specifically focused on rosuvastatin and did not explore the potential unique influence mechanisms of other statins. Further research is needed to investigate the specific effects of different statins on the gut microbiota and their lipid-lowering efficacy. Fourth, we did not have detailed food intake information. In the future, we are looking forward to larger sample cohort studies to confirm our findings. Moreover, animal and cell experiments could be conducted to explore the possible regulatory mechanism. As the interaction mechanism between gut microbiota and statins continues to be studied in depth, it will provide new insights for the precise treatment of hypercholesterolemia in the future.

## 5 Conclusion

In summary, our study shows chenodeoxycholic acid-3-β-D-glucuronide, 1-methylnicotinamide and acetoacetate in stool samples of the US group were significantly higher than those of the BS group. The findings suggest that metabolites related to short-chain fatty acids and secondary bile acids in the gut are expected to be biomarkers indicating the efficacy of statins. Further researches are needed.

## Data availability statement

The raw data supporting the conclusions of this article will be made available by the authors, without undue reservation.

## Ethics statement

The study was approved by the Ethics Committee of the Institute of Biomedicine, Peking University First Hospital, China. The studies were conducted in accordance with the local legislation and institutional requirements. Written informed consent for participation in this study was provided by the participants’ legal guardians/next of kin.

## Author contributions

LH: Data curation, Formal analysis, Funding acquisition, Investigation, Methodology, Software, Validation, Writing – review & editing. BH: Formal analysis, Software, Writing – original draft. LZ: Data curation, Methodology, Writing – review & editing. YH: Formal analysis, Validation, Writing – original draft. YLZ: Validation, Writing – review & editing. RZ: Validation, Writing – review & editing. HY: Validation, Writing – review & editing. DL: Validation, Writing – review & editing. XW: Writing – review & editing. OL: Writing – review & editing. YG: Writing – review & editing. YZ: Writing – review & editing. CL: Writing – review & editing. JL: Funding acquisition, Methodology, Project administration, Writing – review & editing.
